# Early calf segregation enables development of bovine tuberculosis-free replacement stock in a highly infected dairy herd: a preliminary study in Ethiopia

**DOI:** 10.3389/fvets.2025.1551065

**Published:** 2025-03-19

**Authors:** Matios Lakew, Biniam Tadesse, Wegene Bedada, Bayeta Senbata Wakjira, Getnet Abie Mekonnen, Tesfaye Rufael Chibssa, Hagos Ashenafi, Gobena Ameni, Andrew J. K. Conlan, Douwe Bakker, Balako Gumi, Vivek Kapur

**Affiliations:** ^1^Aklilu Lemma Institute of Pathobiology, Addis Ababa University, Addis Ababa, Ethiopia; ^2^Animal Health Institute, Sebeta, Ethiopia; ^3^Department of Veterinary Medicine, College of Agriculture and Veterinary Medicine, United Arab Emirates University, Al Ain, United Arab Emirates; ^4^Disease Dynamics Unit, Department of Veterinary Medicine, University of Cambridge, Cambridge, United Kingdom; ^5^Independent Researcher and Technical Consultant, Lelystad, Netherlands; ^6^Department of Animal Science, The Pennsylvania State University, University Park, PA, United States; ^7^Huck Institutes of the Life Sciences, The Pennsylvania State University, University Park, PA, United States

**Keywords:** bovine tuberculosis control, early calf segregation, dairy cattle, disease surveillance, Ethiopia, replacement stock, test-and-segregation

## Abstract

Bovine tuberculosis (bTB) severely impacts Ethiopia’s growing dairy sector, where test-and-cull control methods are economically unfeasible, and test-and-segregation is impractical in herds with very high prevalence. We assessed the feasibility of establishing bTB-free replacement stock through early segregation of calves born to bTB-positive cows. In a two-year longitudinal study on a high-prevalence (98% tuberculin skin test positive) dairy farm, 26 newborn calves were separated from their bTB-positive dams on day five after birth and screened for bTB at 2 to 5 month intervals across eight rounds, with test-positive calves immediately removed from the negative herd. The majority of segregated calves (19 out of 25; 76%; 95% CI: 58–94) remained bTB-test negative through the testing period, with nine uninfected female calves and two males reaching 18 months of age, demonstrating potential for establishing bTB-free breeding stock. However, six calves (24%; 95% CI: 6–42) turned to test positive during the study period. The extended dam-calf contact during the first five days likely contributed to some infections, suggesting that immediate separation and alternative colostrum sources could improve success rates. The addition of interferon gamma release assays in later testing rounds enabled detection of infected animals potentially missed by skin testing alone, highlighting the value of complementary diagnostic approaches for surveillance. These findings provide preliminary evidence that early calf segregation can generate bTB-negative replacement stock from infected herds, and provide a framework for larger-scale studies across different farm settings.

## Introduction

Ethiopia’s dairy sector is rapidly expanding through intensification to meet growing demand for milk and dairy products (1). However, this intensification has been challenged by increasing prevalence of bovine tuberculosis (bTB), particularly in intensive and semi-intensive dairy farms keeping Holstein-Friesian and crossbreed cattle (2). A systematic review estimated the pooled prevalence of bTB in Ethiopia at 5.8% (2), suggesting that approximately 3.8 million cattle may be infected, given the country’s total cattle population of 66 million (3). At 21.6%, the prevalence among Holstein-Friesian cattle was notably higher. Moreover, studies in central Ethiopia have reported herd-level prevalence rates exceeding 50%, with the prevalence at the animal level estimated to be 24–39%, when the Ethiopian standard cutoff for the comparative cervical test (CCT) > 4 mm was applied (4–6). However, applying the more sensitive diagnostic criteria single cervical test (SCT) > 2 mm or CCT > 0 mm as per the WOAH guidelines would have resulted in even higher estimated prevalence rates (7). The high prevalence of the bTB in central Ethiopia presents a significant risk to emerging dairy farms in regional cities, since the unrestricted sale of bTB-positive cattle from the central region could contribute to the spread of the disease to these areas. This poses a significant challenge to the further development of the dairy sector and raises serious public health concerns.

The high prevalence of bTB in intensifying Ethiopian dairy herds poses unique challenges for disease control. While test-and-cull strategies have proven successful in high-income countries (8–10), this approach is economically unfeasible in Ethiopia due to an often high-proportion of test-positive animals and the lack of affordable replacement stock with similar genetic merit for milk production (11). Alternative strategies such as test-and-segregation, which allow continued production from infected animals while preventing disease spread (12–14), are also impractical in herds where nearly all adult cattle test positive. Thus, alternative locally adapted approaches that balance disease control with the economic realities of dairy producers in low and middle income countries (LMICs) are urgently needed.

In herds with high bTB prevalence, historical evidence suggests an alternative approach: immediate segregation of newborn calves from infected dams to establish disease-free young stock (12, 14). This method, developed by Bernhard Bang in the early 20th century, led to successful establishment of bTB-free herds in several high-prevalence settings (14, 15).

Pilot studies in Ethiopia have demonstrated that the use of the conventional test-and-segregation be an option to effectively reduce bTB prevalence in certain settings. One study reported a decline in bTB prevalence from 48 to 1% after four rounds of testing and segregation (16). Similarly, on another government-owned dairy farm, the prevalence decreased from 22.2 to 0% after five rounds of test-and-segregation conducted over a 12-month study period (17).

However, implementing the test-and-segregation method is challenging in herds where almost all adult cattle are infected. In these circumstances, early calf segregation would offer a practical and economical strategy for developing bTB-free replacement stock while maintaining valuable genetic lines for milk production.

This study therefore assessed the feasibility of establishing bTB-free young stock through immediate segregation and separate management of calves born to bTB-positive cows in a high-prevalence Ethiopian dairy herd. Given the limited information available on implementing such approaches under current Ethiopian dairy farming conditions, we conducted a two-year longitudinal study to evaluate calf infection rates, identify critical control points, and assess the potential for generating bTB-free replacement stock.

## Materials and methods

### Study setting and design

A two-year longitudinal study was conducted from May 2022 to June 2024 at the Animal Health Institute in Sebeta, Ethiopia. The study site maintains an intensively housed research dairy herd of 68 Holstein-Friesian crossbred cattle with 98% of adult cattle testing positive for bTB using comparative cervical test (CCT) cutoff >0 mm and 85% at CCT > 4 mm (Supplementary Table 1).

**Figure 1:**
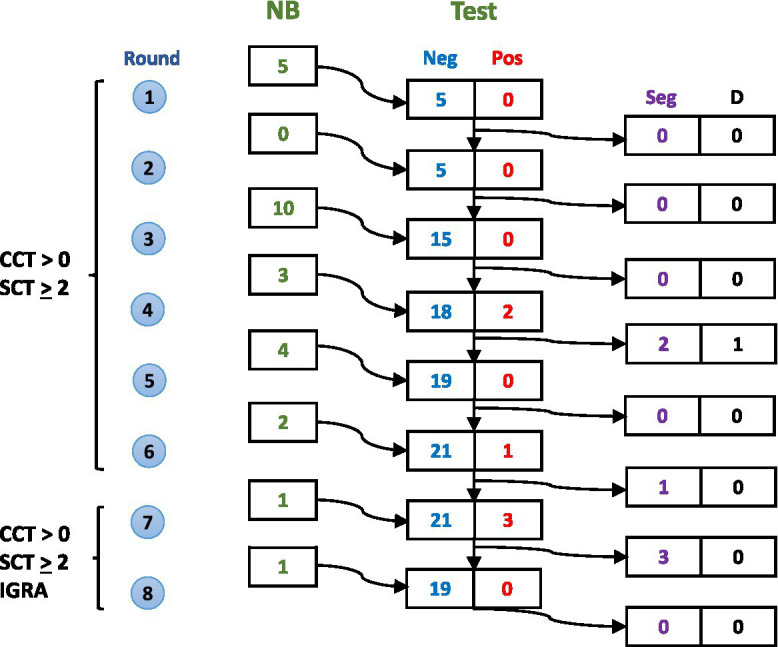
Schematic representation of calf enrollment and testing over eight rounds of surveillance. For each round, numbers show: new calves born (NB), test results (Neg/Pos), animals segregated due to positive results (Seg), and deaths from non-bTB causes (D). Criteria for removing test positive calves evolved from CCT > 0 mm and SCT ≥ 2 mm (rounds 1–6) to include IGRA (rounds 7–8).

The study followed a systematic testing and segregation protocol over eight rounds of surveillance (Figure 1). Newborn calves were enrolled progressively over the study period, with testing conducted every 2–5 months. During the initial six rounds, animals were screened using tuberculin skin tests (CCT > 0 mm and/or single cervical test (SCT) ≥ 2 mm). In the final two rounds, interferon gamma release assay (IGRA) testing was added to increase detection sensitivity. Test-positive animals were immediately removed from the negative group.

### Animals and management

The study included all 26 calves (18 females and 8 males) that were born to bTB-positive dams from the main herd during the study period. Calves remained with their dams for colostrum feeding until day five post-birth, as colostrum from bTB-free animals or a replacer was not available. There was no scientific justification or prior experience for keeping them together for five days; the primary reason was to ensure that the calves received colostrum and milk before being moved to physically separated facilities and managed under strict biosecurity protocols. Calves were fed boiled milk collected from bTB-positive cows, heated to 100°C for ten minutes following established protocols (12). Dedicated animal attendants managed the segregated calves to prevent contact with the infected herd. Housing facilities consisted of separate barns physically isolated from the main herd. Biosecurity measures included the use of protective clothing and equipment for calf attendants, restricted access to calf facilities, separate feeding and watering equipment, regular disinfection, and ensuring prevention of direct and indirect contact with adult cattle. One calf died during the study period due to non-bTB related causes, leaving 25 animals for inclusion in the final analysis. No visible tuberculous lesions were observed during the postmortem examination of the dead animal.

### Diagnostic testing procedures

As shown in Figure 1, the segregated calves underwent eight rounds of tuberculin skin testing at 2–5-month intervals. From the fifth round onwards, testing was supplemented with the IGRA. Test-positive calves were immediately removed from the negative group.

#### Tuberculin skin test

Avian PPD (2,500 IU/dose) and bovine PPD (3,000 IU/dose) (Prionics/ThermoFisher Scientific, Lelystad B.V.) were administered by intradermal injection (0.1 mL each). In adult animals, both PPDs were injected on the same side of the neck, while in smaller calves, injections were administered on opposite sides. Skin thickness was measured before and 72 h post-injection using a manual Irish caliper, with all measurements performed by the same operator. Animals were considered positive if they showed either a CCT > 0 mm or SCT ≥ 2 mm following current guidelines (7). This combined interpretation approach was selected based on previous studies (17).

#### Interferon gamma release assay

Blood samples were collected prior to skin testing for that round in lithium heparin tubes and stimulated in duplicate with PPD A (300 IU/mL), PPD B (250 IU/mL), RPMI1640 media (negative control), and Pokeweed Mitogen (10 μg/mL, positive control). The presence of interferon gamma was measured using the BOVIGAM test kit (product number: 63326, Prionics) according to manufacturer’s instructions. Samples were considered positive when the optical density difference between bovine and avian PPD stimulation was ≥0.1 at 450 nm.

### Data analysis

Test results were summarized using descriptive statistics and visualized using heat maps to display individual animal outcomes over time. Tuberculin skin test and IGRA results were plotted as scatter plots to show the distribution of responses for individual animals. All analyses were conducted using Prism 9 (GraphPad Prism version 9.4.1).

## Results

### Successful generation of bTB-negative replacement stock

**Figure 2:**
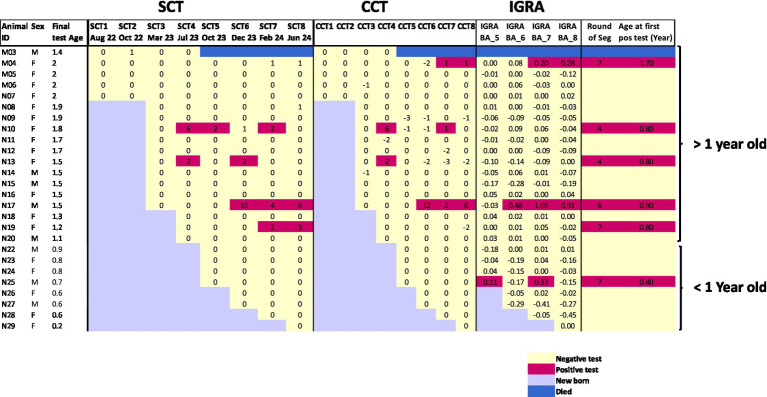
Heat map showing longitudinal test results for individual animals over eight rounds of surveillance. Each row represents one animal (ID and sex shown on left), with final test age in years. Test responses are shown for SCT, CCT, and IGRA. Positive results are highlighted in red-purple, negative results in pale yellow, and new births in light blue. The animal that died between rounds 4–5 is marked in blue. Animals are grouped by age (>1 year and < 1 year old). The right column indicates when positive animals were segregated from the negative herd.

A majority of calves segregated from their bTB-positive dams remained test-negative throughout the study period. Of the 25 calves that completed the study, 19 (76%; 95% CI: 58–94) maintained bTB-negative status across multiple rounds of testing. The calves divided naturally by age, with fourteen animals reaching 1.5 years or older during the study period. Among these older animals, nine females and two males remained uninfected through repeated testing, demonstrating potential as future breeding stock. The remaining test-negative animals were under one year of age at study completion (Figure 2).

### Pattern of test positive reactions over time

Six calves (24%; 95% CI: 6–42) were classified as positive and removed from the negative group during the surveillance period (Supplementary Table 2). The heat map visualization (Figure 2) reveals complex temporal patterns in test responses. The first positive reactors were identified in round 4, where two animals (N10, N13) were detected by both SCT ≥ 2 mm and CCT > 0 mm criteria. However, subsequent testing of these animals showed negative or borderline reactions on skin tests while remaining IGRA negative, suggesting possible false positive initial reactions. A similar pattern was observed with calf N19, which was positive on skin test in round 7 and 8 according to SCT > 2 mm criteria but showed negative CCT and IGRA results.

The introduction of IGRA testing in later rounds led to the detection of three additional animals, including calf N25 which was IGRA positive but skin test negative at less than 6 months of age, when IGRA specificity is known to be reduced. This complex pattern of test results highlights the challenges in interpreting individual positive reactions, particularly in young animals and when using multiple testing approaches with different sensitivity and specificity characteristics.

The testing pattern of calf M04 provides particularly important insights into test performance and potential desensitization effects. This animal showed no reactivity on SCT throughout testing but developed a borderline CCT response and clear IGRA positivity in rounds 7 and 8 (Figure 2). This pattern, where the bovine PPD response appears suppressed, is consistent with previously reported skin test desensitization effects in frequently tested animals. The case highlights both the limitations of relying solely on skin tests for surveillance and the value of IGRA as a complementary test that is not affected by repeated skin testing.

**Figure 3:**
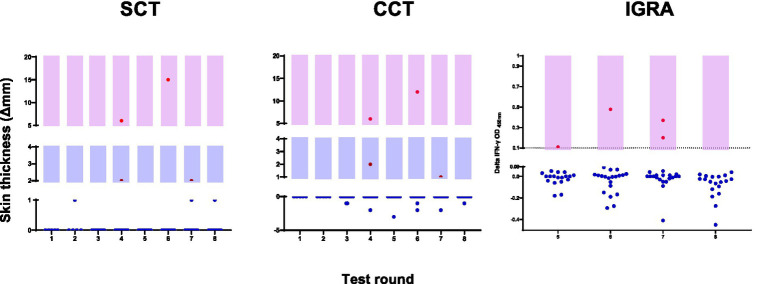
Distribution of individual animal test responses across eight rounds of surveillance. Left panel shows SCT responses, middle panel shows CCT responses, and right panel shows IGRA results. Red dots indicate positive responses based on cutoff values (SCT ≥ 2 mm, CCT > 0 mm, IGRA PPDb-PPDa ≥0.1 OD). Blue dots represent negative test responses. Light purple shading indicates the range of positive values for each test. IGRA testing was implemented from round 5 onwards.

The distribution of individual animal responses across testing rounds (Figure 3) shows the relatively rare occurrence of positive reactions in this group. As summarized in Table 1, of the animals classified as positive, five were identified using skin test criteria (CCT > 0 mm and/or SCT ≥ 2 mm). Of these, three were detected by both cutoffs, while one each was identified by CCT > 0 mm or SCT ≥ 2 mm alone. The less sensitive CCT > 4 mm cutoff would have identified only two of these animals.

**Table 1:** Summary of skin test and IGRA results by test round.

Test round	Round 1	Round 2	Round 3	Round 4	Round 5	Round 6	Round 7	Round 8
No. of tested animals	5	5	15	18	19	21	21	19
Test	Number of test positives (%)							
SCT ≥ 2 mm	0 (0)	0 (0)	0 (0)	2 (11)	0 (0)	1 (5)	1 (5)	0 (0)
CCT > 0 mm	0 (0)	0 (0)	0 (0)	2 (11)	0 (0)	1 (5)	1 (5)	0 (0)
CCT > 4 mm	0 (0)	0 (0)	0 (0)	1 (6)	0 (0)	1 (5)	0 (0)	0 (0)
IGRA PPDb-PPDa	-	-	-	-	1 (5)	1 (5)	2 (10)	0 (0)

Finally, to evaluate whether dam infection status influenced calf outcomes, we analyzed the test responses of the dams throughout the study period (Supplementary Figure 1). All dams showed evidence of bTB infection, with varying levels of skin test and IGRA responses (CCT ranging from 1–45 mm, IGRA BA from 0.15–2.85). Notably, there was no clear association between dam test responses and calf outcomes. Among six dams that each produced two calves during the study period, three had discordant outcomes with one calf remaining negative while the other became test-positive. Even dams with strong positive responses (e.g., CCT > 40 mm) produced calves that remained test-negative, while some dams with moderate reactions had calves that converted to positive. These patterns suggest that post-birth exposure rather than in-utero transmission may be the primary risk factor for calf infection, supporting the potential value of immediate separation strategies.

## Discussion

The high prevalence of bTB in Ethiopian dairy herds, particularly in central regions where rates exceed 50% at the herd level (4, 6), makes conventional control measures impractical. In such settings, test-and-cull approaches would require removing nearly all productive animals, while test-and-segregation becomes pointless when most adult cattle are infected. Our study demonstrates that early segregation of calves born to bTB-positive dams can offer a practical alternative, with 76% of segregated calves remaining test-negative over a two-year period. Most importantly, nine females and two males reached breeding age without evidence of infection, suggesting the feasibility of establishing bTB-free replacement stock while maintaining valuable genetic lines and production capacity. This approach builds on the historical success of the Bang method, developed for high-prevalence settings in the early 20th century (12, 14). Bang’s approach led to the establishment of multiple bTB-free herds through systematic calf segregation and careful management (14, 15). Our findings suggest similar success is possible in modern Ethiopian dairy systems, offering a path forward that balances disease control with the economic realities of the developing dairy sector (1). While recent pilot studies have shown that conventional test-and-segregation can reduce bTB prevalence in some Ethiopian settings (16, 17), the findings of this study demonstrate that early calf segregation may accelerate building a replacement herd, and might be more suitable for herds where adult cattle prevalence approaches 100%.

Our findings highlight several critical control points for successful implementation of calf segregation programs. Most notably, allowing calves to remain with their dams for five days post-birth likely contributed to some infection transmission. While newborn calves received colostrum during this period, the extended contact with highly infected dams presents clear transmission risks through multiple routes including aerosol, direct contact, and milk consumption (18–20). Though rare, there is also potential for congenital tuberculosis when the disease affects the dam’s reproductive tract (18, 21–23). The observation that highly reactive dams could produce uninfected calves, and that the same dam could have different outcomes with different calves, further supports focusing on post-birth management rather than dam selection. This suggests that successful programs should prioritize early calf separation and biosecurity protocols. Future programs should aim for immediate post-birth calf separation without suckling colostrum from the infected dam, using colostrum alternatives such as stored colostrum from known bTB-free cows, properly heat-treated/pasteurized colostrum, or commercial colostrum replacers (24–26).

The testing patterns observed in our study also reveal important considerations for surveillance strategies. The observation of potentially false positive skin test reactions in some calves (N10, N13, N19), and the evidence of skin test desensitization in others (M04), suggests that frequent testing could complicate accurate diagnosis. Considering the potential desensitization following repeated tuberculin skin tests performed at short intervals, it may be important to conduct the skin test at longer intervals, for example at once every four months. However, further research will be needed to determine the most appropriate interval. Calves born in between rounds of testing can be segregated and housed in a separate barn until the next/subsequent test is conducted, and should only be mixed with the negative herd after bTB screening and test negative status. The complementary use of IGRA testing helped resolve some of these cases, though interpretation remains challenging in young calves where test specificity may be reduced. These findings suggest that surveillance programs should carefully balance testing frequency against potential desensitization effects, while considering age-appropriate interpretation of test results. It will be important to explore additional testing methods to enhance the detection of infected calves as early as possible.

The successful implementation of calf segregation programs requires careful attention to practical requirements and infrastructure. Our study was conducted in a government facility with veterinary supervision and dedicated staff, conditions that may be challenging to replicate in private dairy settings. Essential requirements include separate housing facilities with adequate ventilation, dedicated animal attendants, and strict biosecurity protocols to prevent both direct and indirect contact between segregated calves and the infected herd. The need to handle milk safely, through proper heat treatment (12), adds another layer of management complexity. Additionally, as newly segregated calves may have unknown infection status, separate compartments are needed to house them until testing confirms their status, further increasing facility requirements.

Economic considerations also influence feasibility. While this approach allows retention of valuable genetic lines and continued milk production from infected cows, it requires significant investment in facilities, staff, and testing programs. However, compared to test-and-cull strategies that would require immediate replacement of nearly all productive animals in high-prevalence herds (11), the gradual development of a bTB-free replacement herd may be more economically viable for Ethiopian dairy farmers.

This study was conducted at a government research institute, where a previously constructed barn was used for the segregation of newborn calves. As a result, the cost incurred was for recruiting separate animal attendants and reagent for bTB screening. Therefore, the total cost of this study using the early calf segregation approach was not estimated, as it would not provide comprehensive information. Based on our previous experience with bTB control through the test-and-segregation method, costs were primarily associated with the bTB screening tests (including reagents and staff costs), the cost of dedicated animal handlers, and the major expense being the construction of a new barn to keep the segregated animals. From our previous cost estimations for the test-and-segregation approach (17), the per capita/per head cost was estimated at US$ 52.

Recent evidence that BCG vaccination significantly reduces bTB transmission in cattle (27) suggests that combining vaccination with calf segregation could substantially improve the success of control programs. The demonstrated high indirect efficacy of BCG in reducing transmission could help protect segregated calves from residual exposure risks, while its direct protective effect would add another layer of security to the segregation strategy. Successful scaling of this approach would require training of farm owners and staff in biosecurity protocols, along with regular veterinary supervision and systematic testing programs, but the addition of BCG vaccination could make the strategy more robust and feasible across diverse farming conditions.

### Study limitations and future directions

This study was conducted over a relatively short period at a single government-owned facility where optimal conditions for calf segregation could be maintained. The findings may not fully translate to private dairy farm settings where infrastructure, staffing, and biosecurity protocols may be more challenging to implement. Some calves introduced in later rounds were tested only a few times, and these animals require continued monitoring as they age. Moreover, our practice of keeping calves with their dams for the first five days after birth because of inability to source or appropriately treat the colostrum likely substantially increased infection risk, suggesting that immediate separation as per the original Bang protocols should be evaluated in future studies.

Larger-scale trials of the early separation strategy across different farm settings will be required to assess its practical applicability in those situations. Additionally, further research is needed to refine the approach and address any challenges that may arise under diverse management conditions. These studies should evaluate immediate post-birth separation protocols, alternative colostrum management strategies, and optimal testing schedules that balance surveillance needs against desensitization effects. The recent evidence for BCG vaccine efficacy in reducing transmission also suggests exciting possibilities for combining vaccination with calf segregation programs. Long-term studies of such integrated approaches could help define best practices for establishing bTB-free replacement herds while maintaining valuable genetic resources in high-prevalence settings.

## Data Availability

The original contributions presented in the study are included in the article/Supplementary material, further inquiries can be directed to the corresponding authors.
