# Supplementary material for: Early calf segregation enables development of bovine tuberculosis-free replacement stock in a highly infected dairy herd: a preliminary study in Ethiopia
Source: Front Vet Sci. 2025 Mar 19;12:1551065. doi: 10.3389/fvets.2025.1551065 (PMC11963379; doi:10.3389/fvets.2025.1551065)
Supplement: Supplementary file 1 [file Data_Sheet_1.DOCX]

Early calf segregation enables development of bovine tuberculosis-free replacement stock in a highly infected dairy herd: a preliminary study in Ethiopia

Matios Lakew^1,2*^, Biniam Tadesse^2^, Wegene Bedada^2^, Bayeta Senbeta^2^, Getnet Abie Mekonnen^2^, Tesfaye Rufael Chibssa^2^, Hagos Ashenafi^1^, Gobena Ameni^1,3^, Andrew J.K. Conlan^4^ Douwe Bakker^5^, Balako Gumi^1*^ and Vivek Kapur^6, 7*^

**Affiliations:**

^1^Aklilu Lemma Institute of Pathobiology, Addis Ababa University, P.O. Box 1176, Addis Ababa, Ethiopia. ^2^Animal Health Institute, P.O. Box 04, Sebeta, Ethiopia. ^3^Department of Veterinary Medicine, College of Agriculture and Veterinary Medicine, United Arab Emirates University, PO Box 15551, Al Ain, United Arab Emirates. ^4^Disease Dynamics Unit, Department of Veterinary Medicine; University of Cambridge, United Kingdom.^5^Independent Researcher and Technical Consultant, Lelystad, Netherlands. ^6^Department of Animal Science, The Pennsylvania State University, University Park, PA, United States. ^7^Huck Institutes of the Life Sciences, The Pennsylvania State University, University Park, PA, USA.

*Corresponding authors: Matios Lakew: matioslakew@gmail.com, Balako Gumi: balako.gumi@aau.edu.et, Vivek Kapur: vkapur@psu.edu

Supplementary Table 1: Summary of previous bTB test results for 68 cows

| **Test type** | **Number (%) positive** |
| --- | --- |
| CCT > 0, B > A | 67 (98.5) |
| CCT > 2 | 60 (88.2) |
| CCT > 4 | 58 (85.3 |
| SCT > 2 | 67 (98.5) |
| IGRA | 65 (95.6) |

Supplementary Table 2: Additional information on segregated test positive calves

| **Animal ID** | **Round included in the study** | **Round detected as test positive** | **Age at first positive test (Years)** |
| --- | --- | --- | --- |
| M04 | R-1 | R-7 | 1.7 |
| N10 | R-3 | R-4 | 0.8 |
| N13 | R-3 | R-4 | 0.8 |
| N17 | R-3 | R-6 | 0.9 |
| N19 | R-4 | R-7 | 0.8 |
| N25 | R-5 | R-7 | 0.4 |

Supplementary Figure 1: The test results of dams that gave birth to calves. During the study period, twenty bTB positive cows gave birth to a total of twenty-six calves, with six of these cows each giving birth to two calves. The test results for cows that gave birth to calves that remained test-negative and those whose calves tested positive for bTB did not differ. In three instances, cows that gave birth to two calves had one calf test negative while the other tested positive. The heat map shows the skin test (comparative cervical test, CCT) and interferon gamma release assay (IGRA) test results for the cows before the study (up to 2022), during the study (2023), and at the end of the study (May 2024)." The cows that either died (D) or were not tested (NT) are also indicated.
